# Supplementary figures and images for: TTLL12 is required for primary ciliary axoneme formation in polarized epithelial cells
Source: EMBO Rep. 2023 Dec 19;25(1):15. doi: 10.1038/s44319-023-00005-5 (PMC10883266; doi:10.1038/s44319-023-00005-5)

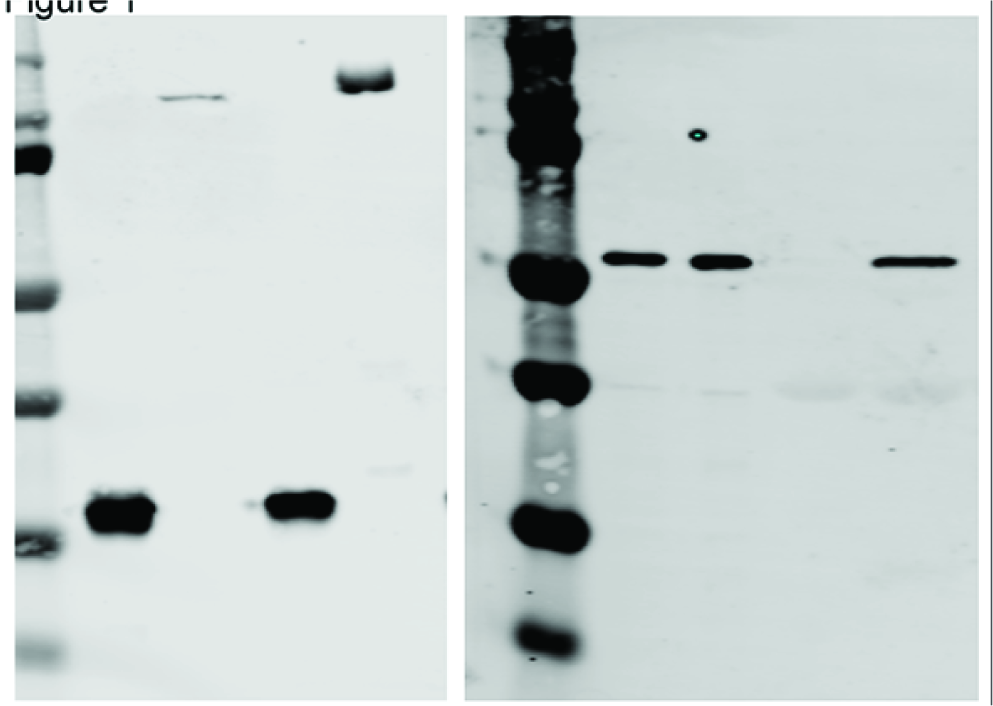

Supplement: Supplementary file 6 — Source Data Fig. 1 [file 44319_2023_5_MOESM6_ESM.zip › Figure 1 source/gells figure 1.tif]

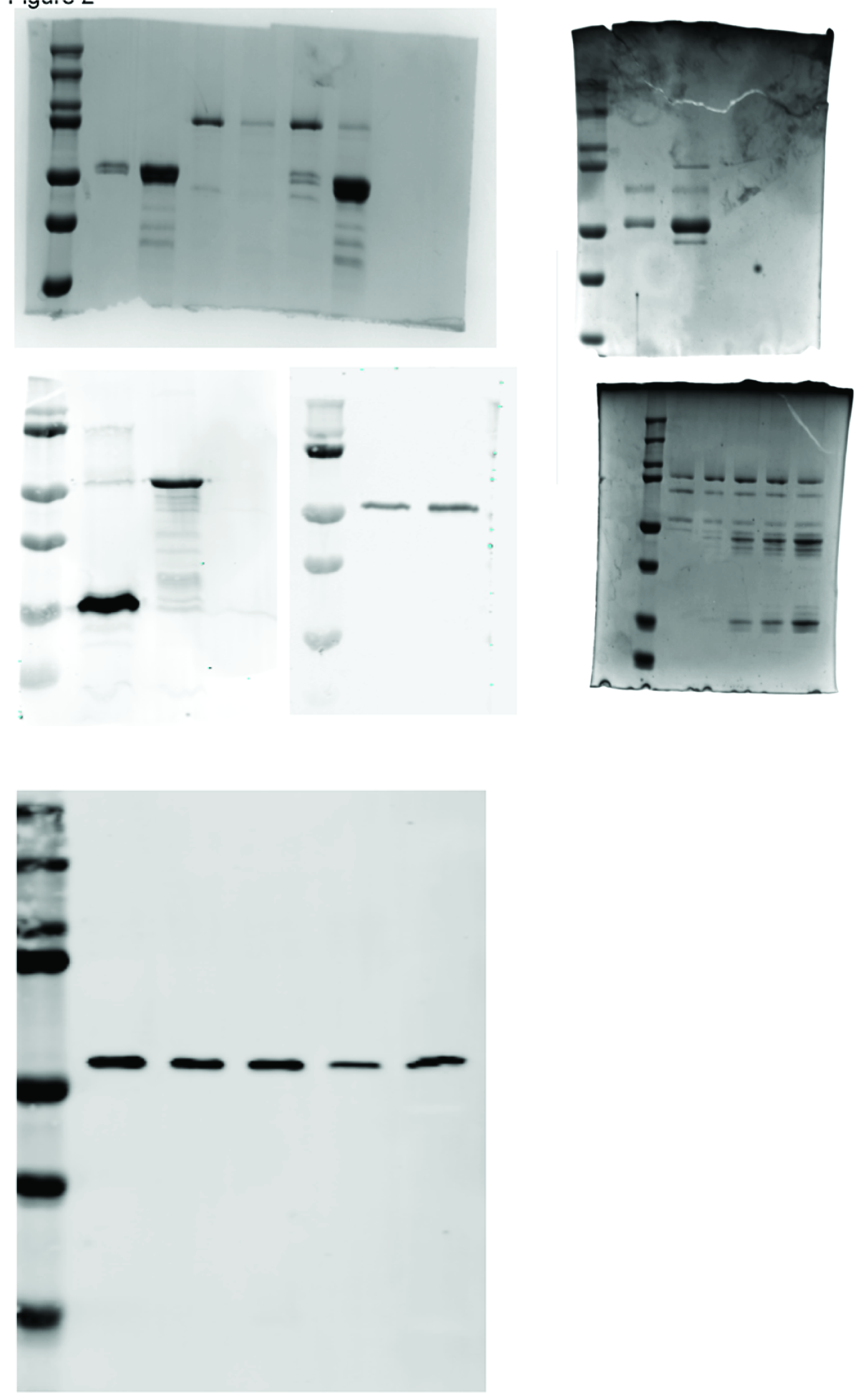

Supplement: Supplementary file 7 — Source Data Fig. 2 [file 44319_2023_5_MOESM7_ESM.zip › Figure 2 source/gells figure 2.tif]

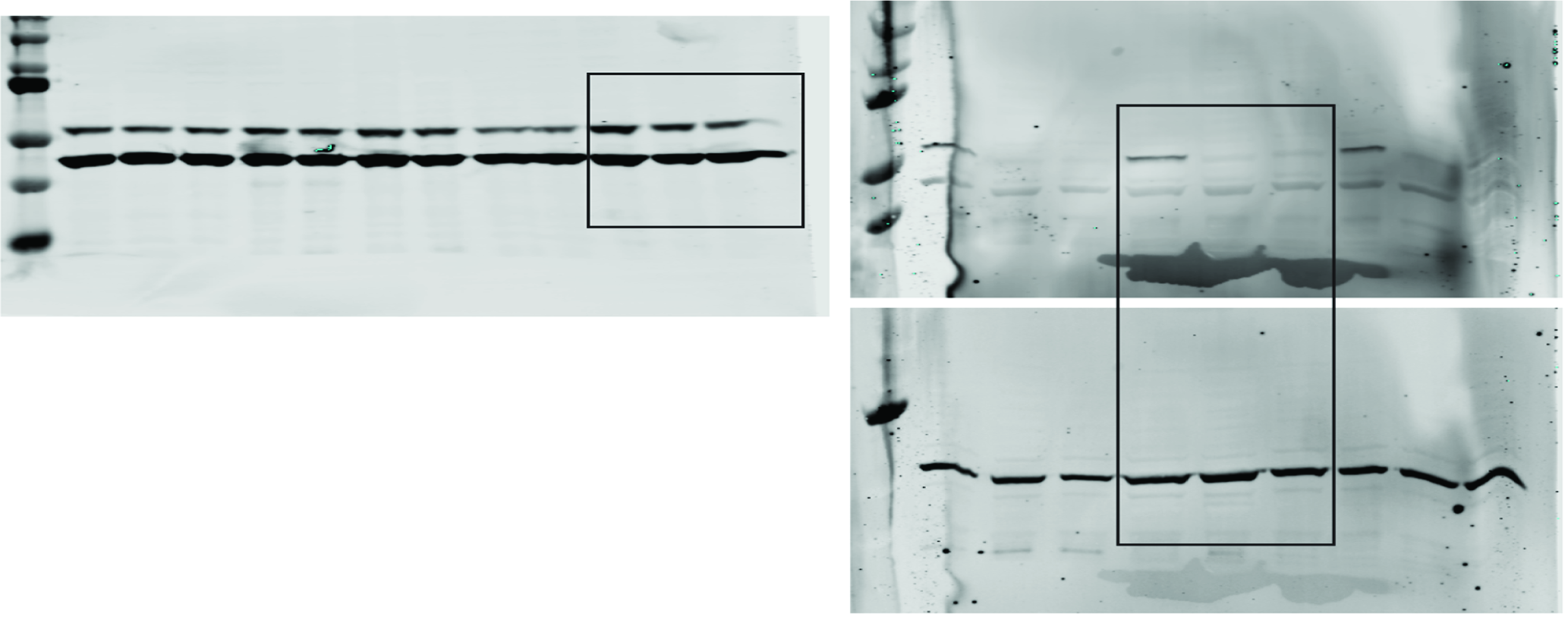

Supplement: Supplementary file 8 — Source Data Fig. 3 [file 44319_2023_5_MOESM8_ESM.zip › Figure 3 source/gells figure 3.tif]

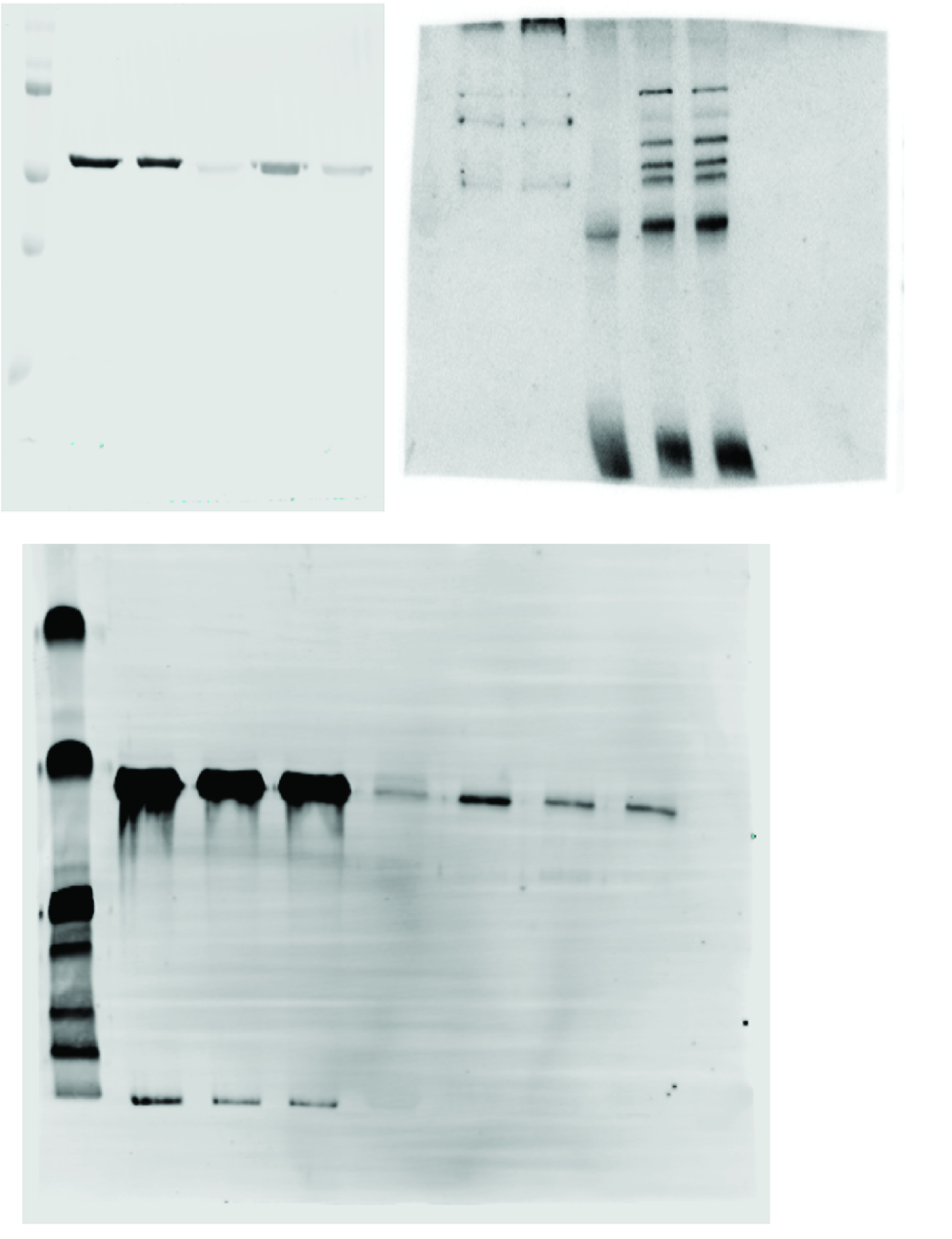

Supplement: Supplementary file 12 — Source Data Fig. 7 [file 44319_2023_5_MOESM12_ESM.zip › Figure 7 source/gells figure 7.tif]
