# Supplementary material for: TTLL12 is required for primary ciliary axoneme formation in polarized epithelial cells
Source: EMBO Rep. 2023 Dec 19;25(1):15. doi: 10.1038/s44319-023-00005-5 (PMC10883266; doi:10.1038/s44319-023-00005-5)
Supplement: Supplementary file 13 — Expanded View Figures [file 44319_2023_5_MOESM13_ESM.pdf]

Expanded View Figures

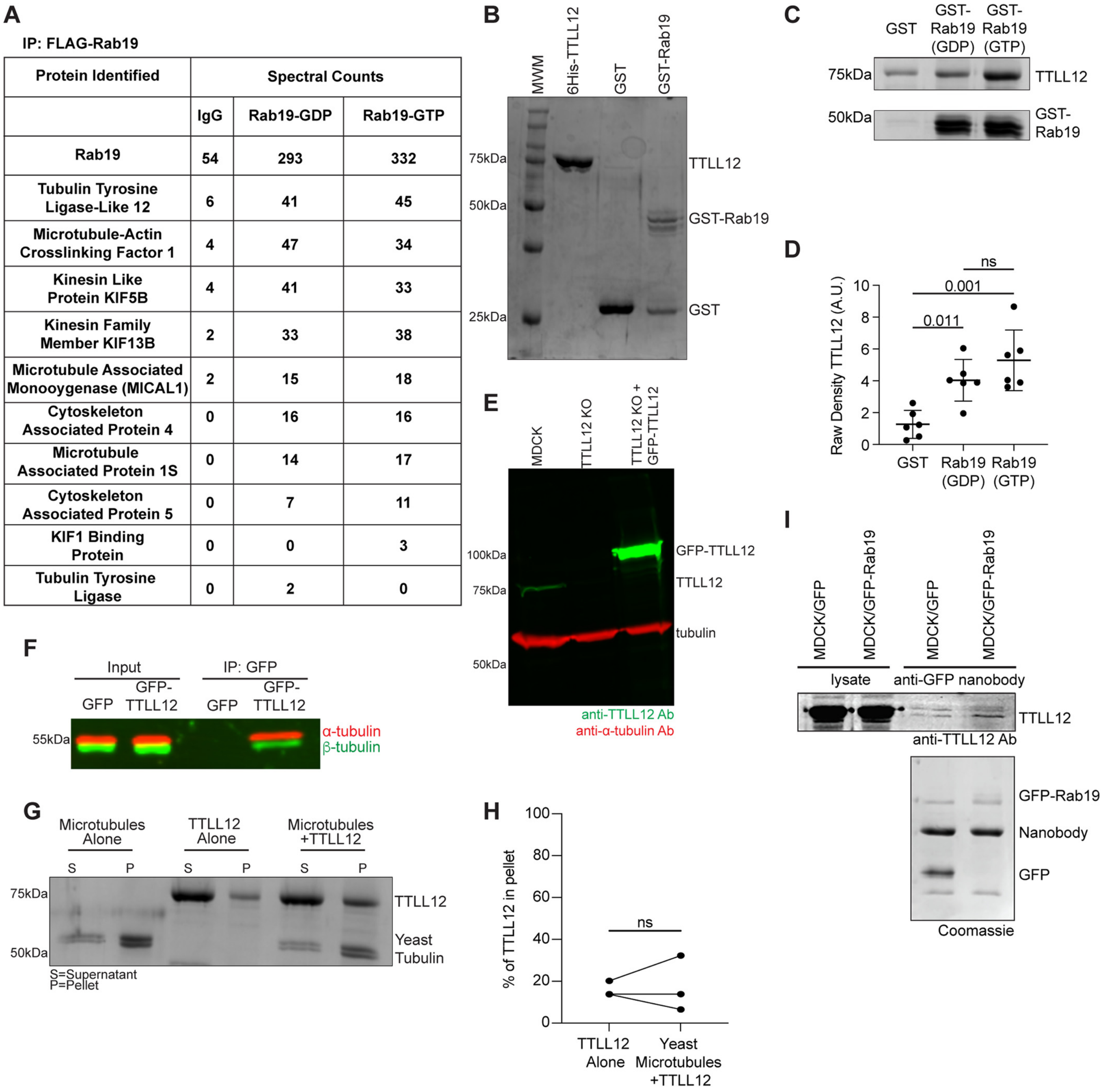

A

IP: FLAG-Rab19

Protein Identified	Spectral Counts		
	IgG	Rab19-GDP	Rab19-GTP
Rab19	54	293	332
Tubulin Tyrosine Ligase-Like 12	6	41	45
Microtubule-Actin Crosslinking Factor 1	4	47	34
Kinesin Like Protein KIF5B	4	41	33
Kinesin Family Member KIF13B	2	33	38
Microtubule Associated Monooygenase (MICAL1)	2	15	18
Cytoskeleton Associated Protein 4	0	16	16
Microtubule Associated Protein 1S	0	14	17
Cytoskeleton Associated Protein 5	0	7	11
KIF1 Binding Protein	0	0	3
Tubulin Tyrosine Ligase	0	2	0

Figure EV1. (**A**) Spectral counts of microtubule-related candidate proteins identified through co-immunoprecipitation/mass spectrometry on FLAG-Rab19 from Jewett et al (2021). (**B**) Coomassie blue-stained gel with recombinant purified 6His-TTLL12, GST, and GST-Rab19. (**C**) Binding assay with recombinant GST-Rab19, locked with either GDP or GMP-PNP (GTP), and recombinant 6His-TTLL12 followed by Coomassie blue staining. (**D**) Quantification of TTLL12 band intensity in (**B**). Graph shows mean ± SD derived from three independent experiments. Student's *t* test (two-tailed) was used for statistical analysis. (**E**) Western blot of MDCK WT, TTLL12 KO, and TTLL12 KO cells stably expressing GFP-TTLL12 for TTLL12 (green band). (**F**) Immunoprecipitation of GPF-TTLL12 followed by western blot for α- and β-tubulin. (**G**) Microtubule co-precipitation assay. Taxol-stabilized yeast microtubules were mixed with recombinant 6His-TTLL12 and pelleted by centrifugation. The supernatant and pellet were separated and run on a gel followed by Coomassie blue staining. (**H**) Quantification of TTLL12 in the pellet with or without microtubules from (**G**). Graph shows mean ± SD from three independent experiments. Student's *t* test (two-tailed) was used for statistical analysis. (**I**) Immunoprecipitation of GPF-Rab19 or GFP-only from MDCK cells followed by western blot for TTLL12.

© The Author(s)

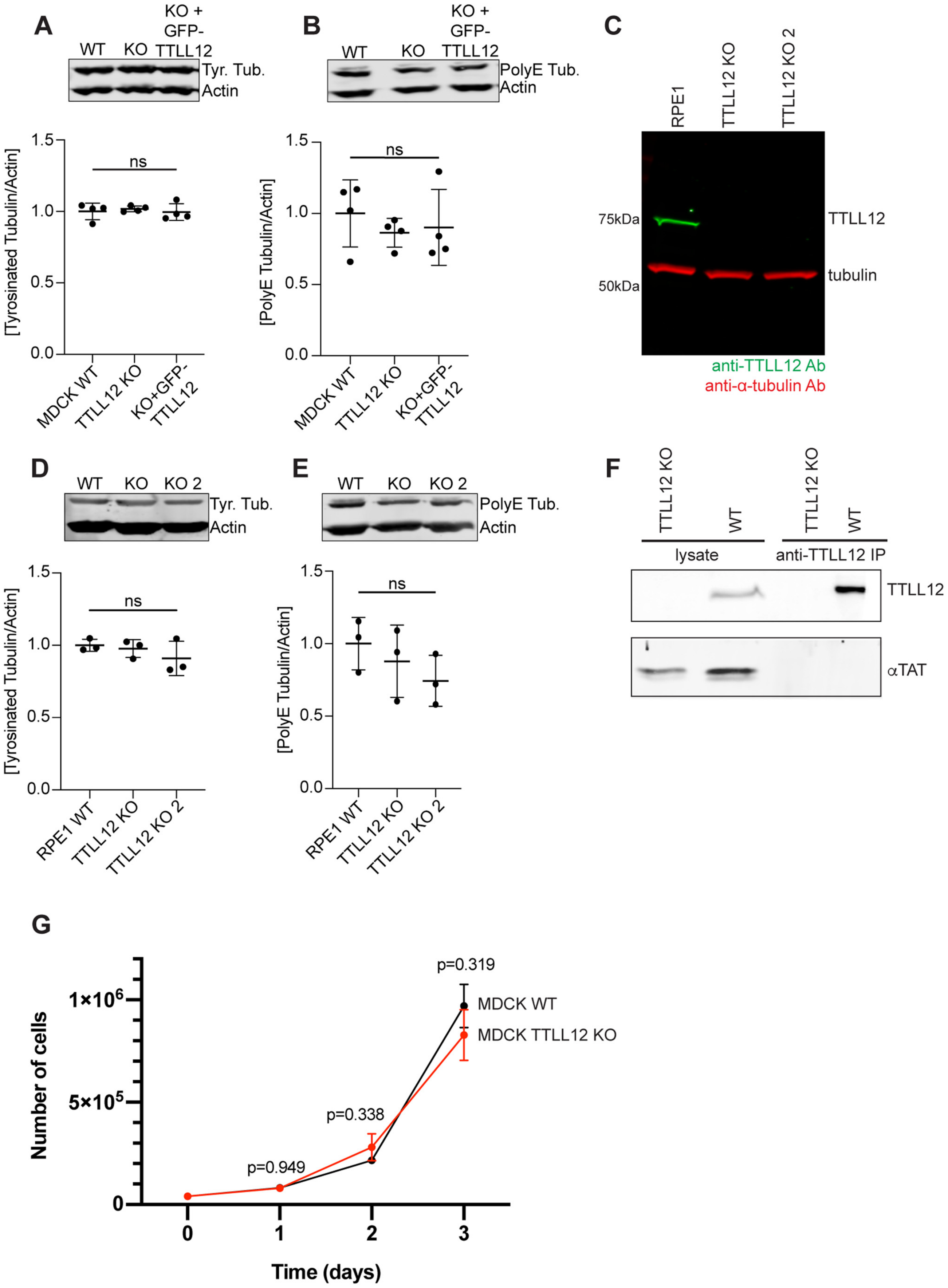
A
WT
KO
KO + GFP-TTLL12
Tyr. Tub.
Actin
[Tyrosinated Tubulin/Actin]
ns
MDCK WT
TTLL12 KO
KO+GFP-TTLL12
B
PolyE Tub.
[PolyE Tubulin/Actin]
C
RPE1
TTLL12 KO
TTLL12 KO 2
75kDa
50kDa
TTLL12
tubulin
anti-TTLL12 Ab
anti-α-tubulin Ab
D
KO 2
RPE1 WT
TTLL12 KO 2
E
F
lysate
anti-TTLL12 IP
αTAT
G
Number of cells
Time (days)
p=0.949
p=0.338
p=0.319
MDCK WT
MDCK TTLL12 KO

◀ **Figure EV2.** (**A**) Representative western blot and quantification of tyrosinated α-tubulin in MDCK WT, TTLL12 KO, and TTLL12 KO + GFP-TTLL12 cells. Graph shows mean ± SD derived from four independent experiments. Student's *t* test (two-tailed) was used for statistical analysis. (**B**) Representative western blot and quantification of polyglutamylated tubulin in MDCK WT, TTLL12 KO, and TTLL12 KO + GFP-TTLL12 cells. Graph shows mean ± SD derived from four independent experiments. Student's *t* test (two-tailed) was used for statistical analysis. (**C**) Western blot of RPE1 WT, TTLL12 KO, and TTLL12 KO 2 cells for TTLL12 (green band). (**D**) Representative western blot and quantification of tyrosinated α-tubulin in RPE1 WT, TTLL12 KO, and TTLL12 KO 2 cells. Graph shows mean ± SD derived from three independent experiments. Student's t test (two-tailed) was used for statistical analysis. (**E**) Representative western blot and quantification of polyglutamylated tubulin in RPE1 WT, TTLL12 KO, and TTLL12 KO 2 cells. Graph shows mean ± SD derived from three independent experiments. Student's *t* test (two-tailed) was used for statistical analysis. (**F**) Western blot of TTLL12 precipitate from wild-type or TTLL12 KO RPE1 cells. (**G**) Proliferation analysis of MDCK WT and MDCK TTLL12 KO cells. One-way ANOVA was used for statistical analysis. The data shown are the means and standard deviations derived from three independent experiments.

© The Author(s)

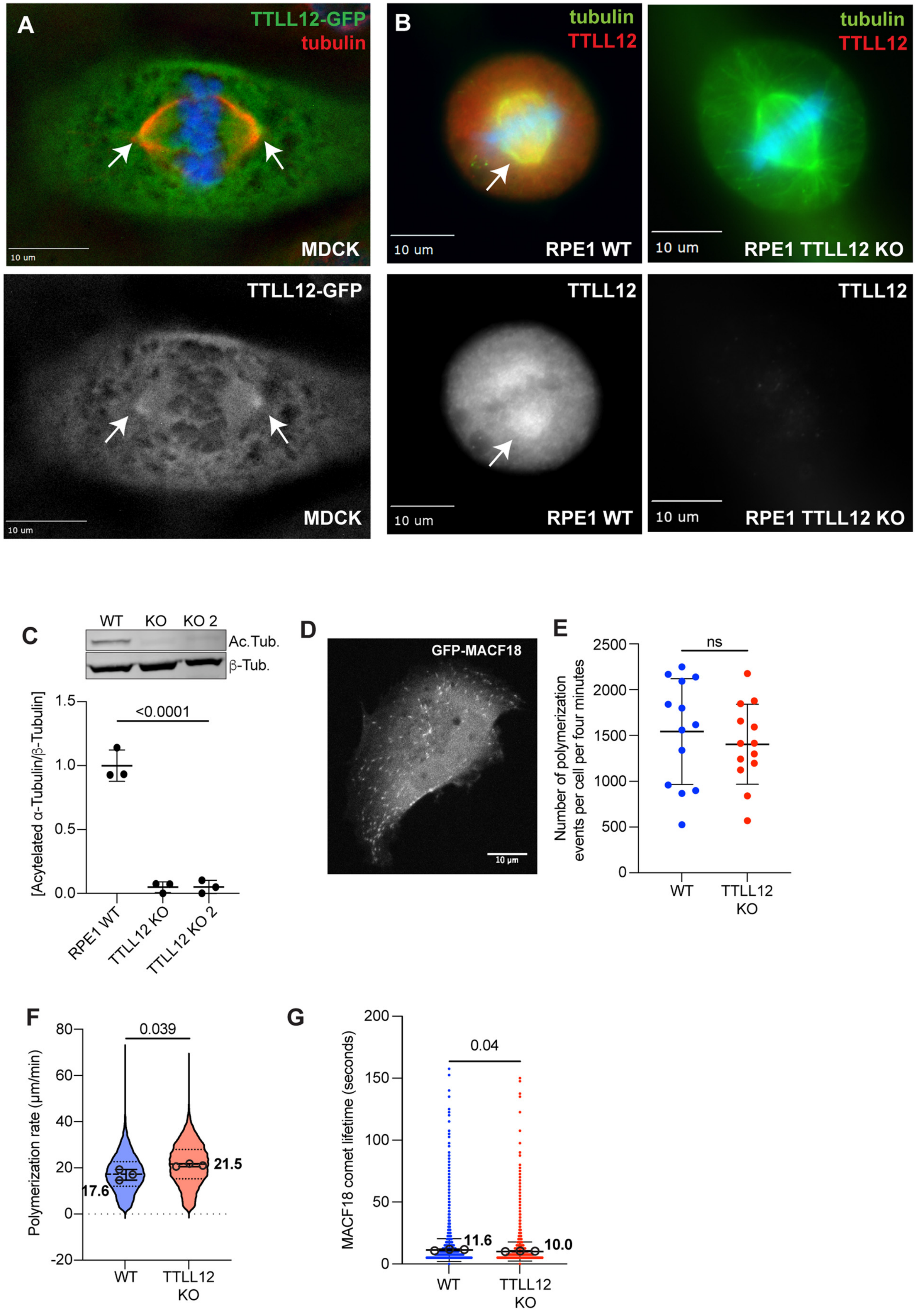
A
TTLL12-GFP
tubulin
MDCK
10 um
TTLL12-GFP
MDCK
10 um
B
tubulin
TTLL12
10 um
RPE1 WT
tubulin
TTLL12
10 um
RPE1 TTLL12 KO
TTLL12
10 um
RPE1 WT
TTLL12
10 um
RPE1 TTLL12 KO
C
WT KO KO 2
Ac.Tub.
β-Tub.
[Acytelated α-Tubulin/β-Tubulin]
<0.0001
RPE1 WT
TTLL12 KO
TTLL12 KO 2
D
GFP-MACF18
10 μm
E
Number of polymerization events per cell per four minutes
ns
WT
TTLL12 KO
F
Polymerization rate (μm/min)
0.039
17.6
21.5
WT
TTLL12 KO
G
MACF18 comet lifetime (seconds)
0.04
11.6
10.0
WT
TTLL12 KO

 © The Author(s)

Figure EV3. (**A**) MDCK cells stably expressing TTLL12-GFP were stained with anti-α-tubulin antibodies. Arrows point to the mitotic spindle. (**B**) WT or TTLL12 KO RPE cells were stained with anti-TTLL12 (green) and anti-α-tubulin (red) antibodies. Arrow points to the mitotic spindle. (**C**) Representative western blot and quantification of acetylated α-tubulin in RPE1 WT, TTLL12 KO, and TTLL12 KO 2 cells. Graph shows mean ± SD derived from three independent experiments. Student's *t* test (two-tailed) was used for statistical analysis. (**D**) Example image of WT RPE1 cell expressing GFP-MACF18 used for live imaging of microtubule polymerization. Scale bar = 5 μm. (**E**) Quantification of the number of microtubule polymerization events that occur in each cell over the course of 4 min. Images were obtained from three independent experiments. $n = 13$ cells for both WT and TTLL12 KO. Graph shows mean ± SD derived from three independent experiments. Student's *t* test (two-tailed) was used for statistical analysis. (**F**) Quantification of microtubule polymerization rates measured from GFP-MACF18 comets. Violin plot represents all microtubules measured ($n = 20{,}427$ for WT, $n = 19{,}525$ for KO). Black circles represent the average polymerization rate from each independent experiment. Graph shows mean ± SD derived from three independent experiments. Student's *t* test (two-tailed) was used for statistical analysis. (**G**) Quantification of MACF18 comet lifetime. Plot represents all microtubules measured ($n = 20{,}427$ for WT, $n = 19{,}525$ for KO). Black circles represent the average polymerization rate from each independent experiment ($N = 3$). *T* test was performed on the means from the experiment. Student's *t* test (two-tailed) was used for statistical analysis.

© The Author(s)

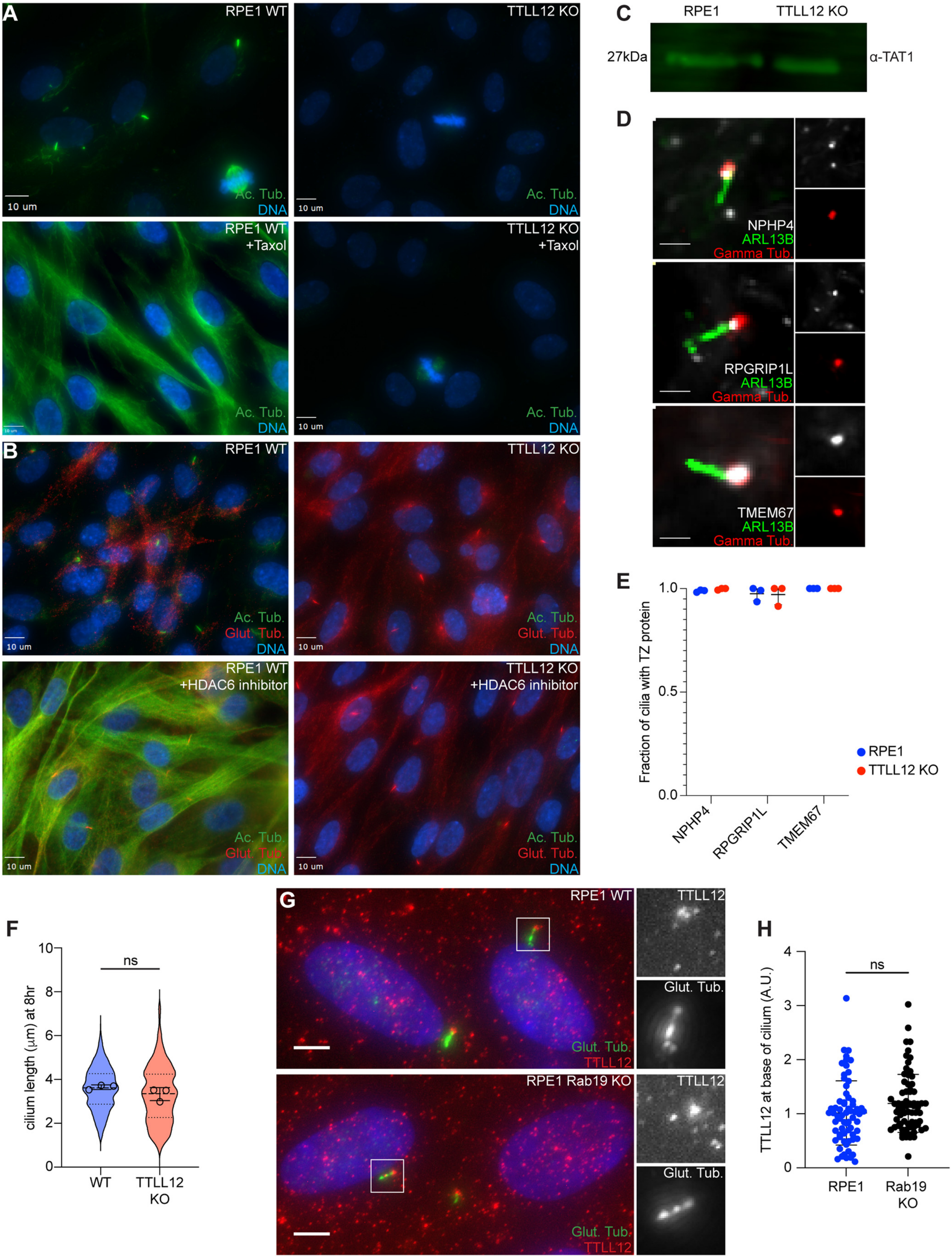
A
RPE1 WT
TTLL12 KO
10 um
Ac. Tub.
DNA
RPE1 WT +Taxol
TTLL12 KO +Taxol
B
RPE1 WT
TTLL12 KO
Ac. Tub.
Glut. Tub.
DNA
RPE1 WT +HDAC6 inhibitor
TTLL12 KO +HDAC6 inhibitor
C
RPE1
TTLL12 KO
27kDa
α-TAT1
D
NPHP4
ARL13B
Gamma Tub.
RPGRIP1L
TMEM67
E
Fraction of cilia with TZ protein
1.0
0.5
0.0
NPHP4
RPGRIP1L
TMEM67
RPE1
TTLL12 KO
F
cilium length (μm) at 8hr
10
8
6
4
2
0
ns
WT
TTLL12 KO
G
RPE1 WT
TTLL12
Glut. Tub.
RPE1 Rab19 KO
H
TTLL12 at base of cilium (A.U.)
4
3
2
1
0
ns
RPE1
Rab19 KO

 © The Author(s)

Figure EV4. (**A**) Representative images of RPE1 WT and TTLL12 KO cells with or without Taxol treatment stained for acetylated tubulin and DNA ($N = 1$). (**B**) Representative images of RPE1 WT and TTLL12 KO cells with or without 1 µm of the pharmacological HDAC6 inhibitor, tubastatin A, and stained for acetylated tubulin, glutamylated tubulin, and DNA ($N = 1$). (**C**) Western blot of αTAT1 in RPE1 WT and TTLL12 KO cells. (**D**) Representative images of transition zone proteins NPHP4, RPGRIP1L, and TMEM67 localized to the basal body (gamma tub.) in TTLL12 KO cells. Scale bars: 5 µm. (**E**) Quantification of RPE1 WT and TTLL12 KO cilia with the respective transition zone proteins from (**G**). Graph shows mean ± SD derived from three independent experiments. (**F**) Quantification of primary cilium length after 8 h of serum starvation. Violin plot represents all primary cilia measured. Black circles represent average cilium length ($n = 538$ for WT, $n = 250$ for KO). Shown are the means and standard deviations derived from three independent experiments. Student's *t* test (two-tailed) was used for statistical analysis. (**G**) Representative images of TTLL12 localization in ciliated RPE1 WT and Rab19 KO cells. Scale bar = 5 µm. (**H**) Quantification of TTLL12 at the base of the primary cilium from G. $N = 2$ and 35 cells were measured in each condition per experiment. Graph shows mean ± SD. Student's *t* test (two-tailed) was used for statistical analysis.

© The Author(s)

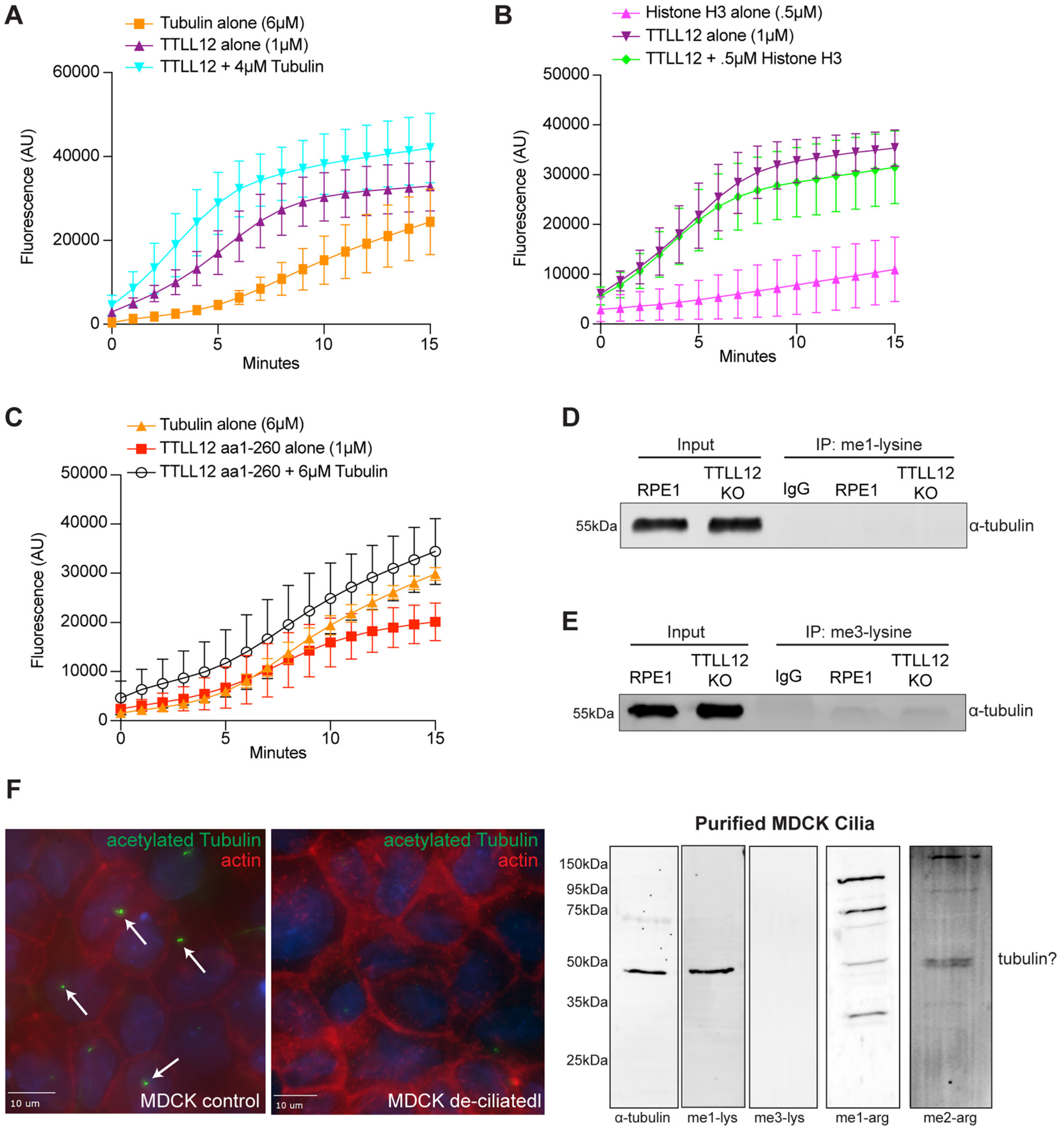

Figure EV5. (**A**) Fluorescence-based assay to measure methyltransferase activity. 1 μM 6His-TTLL12 was incubated with methyl donor SAM and 4 μM porcine brain tubulin and fluorescence was measured over time. Graph shows mean ± SD derived from three independent experiments. (**B**) 1 μM 6His-TTLL12 was incubated with methyl donor SAM and 0.5 μM histone H3 and fluorescence was measured over time. Graph shows mean and variability derived from two independent experiments. (**C**) 1 μM GST-TTLL12 aa1-260 was incubated with methyl donor SAM and 6 μM porcine brain tubulin and fluorescence was measured over time. Graph shows mean and variability derived from two independent experiments. (**D**) Immunoprecipitation of mono-methyl lysine from RPE1 cells followed by western blot for α-tubulin. Left column shows lysates (input) probed for α-tubulin. Right columns show immunoprecipitates probed for α-tubulin. (**E**) Immunoprecipitation of tri-methyl lysine from RPE1 cells followed by western blot for α-tubulin. Left column shows lysates (input) probed for α-tubulin. Right columns show immune-precipitates probed for α-tubulin. (**F**) Representative image of MDCK cells incubated in the presence or absence of high calcium deciliation buffer (images on the left). Arrows point to individual cilia. Blots on right are the purified cilia preparations immunoblotted with anti-α-tubulin, anti-me1K, anti-me3K, anti-me1R, and anti-me2R antibodies.

 © The Author(s)
